# Incidence Rates of Myomectomy-Related Mortality and Venous Thromboembolism in South Korea: A Population-Based Study

**DOI:** 10.3389/fmed.2022.849660

**Published:** 2022-03-10

**Authors:** Jin-Sung Yuk, Myounghwan Kim

**Affiliations:** Department of Obstetrics and Gynecology, School of Medicine, Sanggye Paik Hospital, Inje University, Seoul, South Korea

**Keywords:** fibroids, leiomyoma, mortality, myomectomy, venous thromboembolism

## Abstract

**Background:**

Uterine leiomyomas are the most commonly observed pathologies, with an estimated prevalence of 4. 5–68.6%. We aimed to calculate myomectomy-related mortality and venous thromboembolism incidence rates in the Republic of Korea.

**Methods:**

The data of patients who underwent myomectomy (2009–2018) were obtained from the Health Insurance Review and Assessment Service-National Inpatient Sample. The mortality rate after myomectomy was calculated using the leiomyoma diagnostic codes and myomectomy procedure codes. The incidence rates of venous thromboembolism, deep vein thrombosis, and pulmonary embolism were calculated using their diagnostic codes, with concomitant use of an antithrombotic agent during the same period or within 90 days after myomectomy.

**Results:**

The data of 23,549 women aged 15–55 years who underwent myomectomy were extracted. The myomectomy rate was 14.6 ± 0.1 per 10,000 patients. The average age was 39.39 ± 0.04 years. One patient who underwent myomectomy died; this patient did not have concomitant venous thromboembolism. The post-myomectomy mortality rate was 1.3 ± 0.8 per 10,000 patients. The incidence rates of venous thromboembolism, deep vein thrombosis, and pulmonary embolism after myomectomy were 5.7 ± 1.6 per 10,000 patients, 4.4 ± 1.4 per 10,000 patients, and 2.5 ± 1 per 10,000 patients, respectively. The conversion rate to hysterectomy was 2.9 ± 1.1 per 10,000 patients.

**Conclusion:**

The current mortality rate after myomectomy (0.013%) is substantially lower than that described in previous studies at the turn of the 20th century. The incidence of venous thromboembolism is also considerably lower than that in the general population worldwide.

## Introduction

Uterine leiomyomas, commonly known as fibroids, are among the most common problems encountered by gynecologists, with a prevalence estimated at 4.5–68.6% (1). A research by Baird et al. showed that leiomyomas are discovered by ultrasonography in more than 80% of women of African lineage and in ~70% of white women by the age of 50 years (2). They frequently cause abnormal uterine bleeding and pelvic pain and are implicated in infertility. The contemporary options for symptomatic treatment of leiomyoma comprise expectant management, medical therapy, and surgical treatment or interventional radiology procedures (3).

Uterine leiomyomas are the most common indications for hysterectomy. Many patients with symptomatic leiomyomas want to have the option of future childbearing or simply want to conserve their uterus. For these women, the removal of the leiomyomas alone with reconstruction and preservation of the uterus is a significant option.

Ammussat performed the first myomectomy in 1840 (4). Successful abdominal myomectomy was performed as early as 1845 by Washington and John Atlee. Washington Atlee finally published his experience after 14 abdominal myomectomies, with five deaths in his patient group (5). At the turn of the 20th century, compared with the mortality rate of 6–7% for abdominal hysterectomy, abdominal myomectomy was associated with a mortality rate of 40% (4, 6). Victor Bonney is accredited with advocating and popularizing the procedure in the 1920's (7–9).

Hysterectomy may be required intraoperatively to control bleeding. In the Tufts series described by Iverson et al. (10), conversion to intraoperative hysterectomy was necessary in two of 103 patients who underwent myomectomy (10). In the Yale series described by LaMorte et al. (11), one of the 128 patients required a hysterectomy (11). Approximately 1–4% of open myomectomies are converted to hysterectomy (12, 13). Laparoscopic myomectomies are converted to laparotomy in 8% of the procedures (14). All patients undergoing abdominal or laparoscopic myomectomies should be counseled on the possibility of the above mentioned complications when obtaining consent (14). Furthermore, there are very few studies that elucidate the optimal time for conception after myomectomyA prudent plan is to permit patients to heal for a minimum of few months prior to attempting conception (15). Many studies have reported complications, such as bleeding, infection, visceral injury, bladder and ureter injury, thromboembolism, and uterine rupture in pregnancies occurring after myomectomy (10, 11). Venous thromboembolism (VTE) has long been recognized as a possible complication and leading cause of death in patients undergoing pelvic surgery (16).

Although myomectomy is a common operation in the field of gynecology, there are very few reports on the current mortality rate after myomectomy. Hence, this study aimed to determine the mortality rate after myomectomy in the Republic of Korea using the National Health Insurance Database. Additionally, we determined the VTE rate in this study population.

## Methods

### Study Setting and Participants

Almost all residents of the Republic of Korea are protected by the insurance provided by the National Health Insurance Corporation (NHIC). The Health Insurance Review and Assessment Service (HIRA) decides if the medical fee claimed by healthcare institutions is appropriate and recommends reimbursement by the NHIC in an unbiased manner. Therefore, the HIRA shares a significant part of the NHIC data (17). The HIRA collates the HIRA-National Inpatient Sample (HIRA-NIS) every year, extracting the data of one million people (this includes 10–13% of patients who have been hospitalized at least once and 1% of patients who have never been hospitalized during a single calendar year) enrolled in the health insurance service for medical research. The HIRA patient samples are considered representative of the population, as demonstrated by similar results of studies that used the HIRA-NIS and complete health insurance data (18, 19). This cross-sectional study used the HIRA-NIS 2009–2018 data (Serial numbers: HIRA-NIS-2009-0068/2010-0137/2011-0194/2012-0117/2013-0041/2014-0132/2015-0123/2016-0100/2017-0030/2018-0058) (17). We used the diagnostic codes included in the claims that were classified as per the International Classification of Diseases (ICD-10), the surgical and treatment codes from the Health Insurance Medical Care Expenses (2017–2018 version), and the HIRA Drug Ingredients Codes (drug codes) to select the participants.

### Case Definition

**Figure 1:**
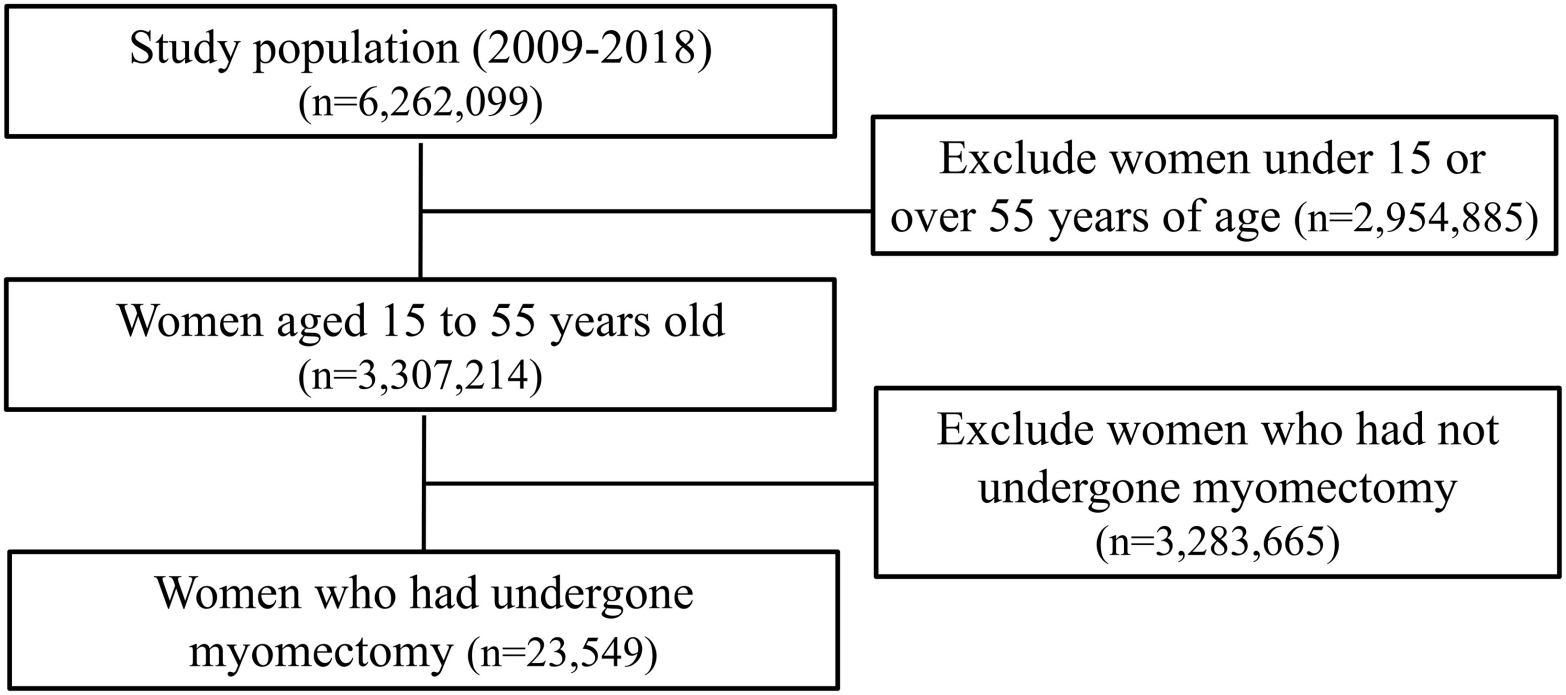
Flowchart showing the selection of the study participants.

Female patients aged 15–55 years who had leiomyoma diagnostic codes simultaneously with myomectomy treatment codes (laparotomic/laparoscopic and not hysteroscopic myomectomies/myomectomy during cesarean section) were enrolled in the study (Figure 1). Patients who died from uterine leiomyomas were defined using a code for death within 90 days of myomectomy; in these cases, if there was a diagnostic code related to cancer in the main or sub-diagnosis on the date of death or if there was a diagnostic code that was not connected to hemorrhage or infection (e.g., trauma due to an accident), “death due to myomectomy” was excluded. In addition, patients with two or more outpatient visits after myomectomy were not defined using the “death due to myomectomy” code.

Patients with venous thromboembolism (VTE), deep vein thrombosis (DVT), and pulmonary embolism (PE) were defined as those who had VTE, DVT, and PE diagnostic codes, respectively, with concomitant use of an antithrombotic agent during the same time period or within 90 days after myomectomy (Table 1). Those diagnosed with VTE, DVT, and PE before or 90 days after myomectomy were excluded from this study. Conversion to hysterectomy during myomectomy was defined as having both myomectomy and hysterectomy codes simultaneously.

**Table 1:** Diagnostic and procedural codes used in this study.

**Names**	**Description**	**Code**
Myomectomy	Simple myomectomy	R4121
	Complex myomectomy	R4122
	Simple abdominal myomectomy	R4124
	Complex abdominal myomectomy	R4127
	Simple laparoscopic myomectomy	R4128
	Complex laparoscopic myomectomy	R4129
VTE	DVT, not otherwise specified	I80.2
	Embolism or thrombosis of lower extremity	I80.3
	Pulmonary thrombosis, pulmonary thromboembolism, or pulmonary infarction	I26
DVT	DVT, not otherwise specified	I80.2
	Embolism or thrombosis of lower extremity	I80.3
PE	Pulmonary thrombosis, pulmonary thromboembolism, or pulmonary infarction	I26
Drug	Aspirin	11070XXX, 11080XXX, 11090XXX, 11100XXX, A0550XXX, 48970XXX, 51790XXX
	DOAC	51140XXX, 61370XXX, 61700XX, 64360XXX
	Fondaparinux	45013XXX
	LMWH	1521XXX, 14023XXX
	UFH	1686XXX
	Warfarin	24910XXX

*DOAC, direct oral anticoagulants; DVT, deep vein thrombosis; LMWH, low molecular weight heparin; PE, pulmonary embolism; VTE, venous thromboembolism; UFH, unfractionated heparin*.

Patients on Medicaid (or an equivalent) for medical insurance were categorized as having a low socioeconomic status (SES). Patients' age was categorized using 5-year intervals. The Charlson comorbidity index (CCI) was verified through diagnostic codes used from January 1 of each year to December 31 of the same year. The CCI was scored in accordance with the method proposed by Quan et al. and categorized into 0, 1, 2, 3, or more (20). The hospitals were classified based on size as clinics, hospitals, general hospitals, and senior general hospitals.

### Statistical Analysis

All statistical analyses in this study were performed using the statistical program R version 3.5.0 (the R Foundation for Statistical Computing, Vienna, Austria). All statistical calculations were performed using two-tailed tests and significance was set at *p* <0.05. All continuous data are expressed as mean ± standard error, and all categorical data are expressed as total numbers (%). Two different sampling weights were used for the data in this study. The weighted *t*-test was utilized for the mean comparison of continuous variables, and the chi-square test was used for the comparison of categorical variables. The weighted mean was used to calculate the mean of the continuous variables, and the weighted ratio was used to calculate the ratio of the continuous variables. If there were missing values, the listwise deletion method was used.

### Ethics

This research study was conducted retrospectively using data obtained for clinical purposes. The Institutional Review Board (IRB) of Sanggye Paik Hospital of Inje University granted an official waiver of ethical approval and informed consent (IRB approval number: SGPAIK 2020-07-006) for this study.

## Results

**Figure 2:**
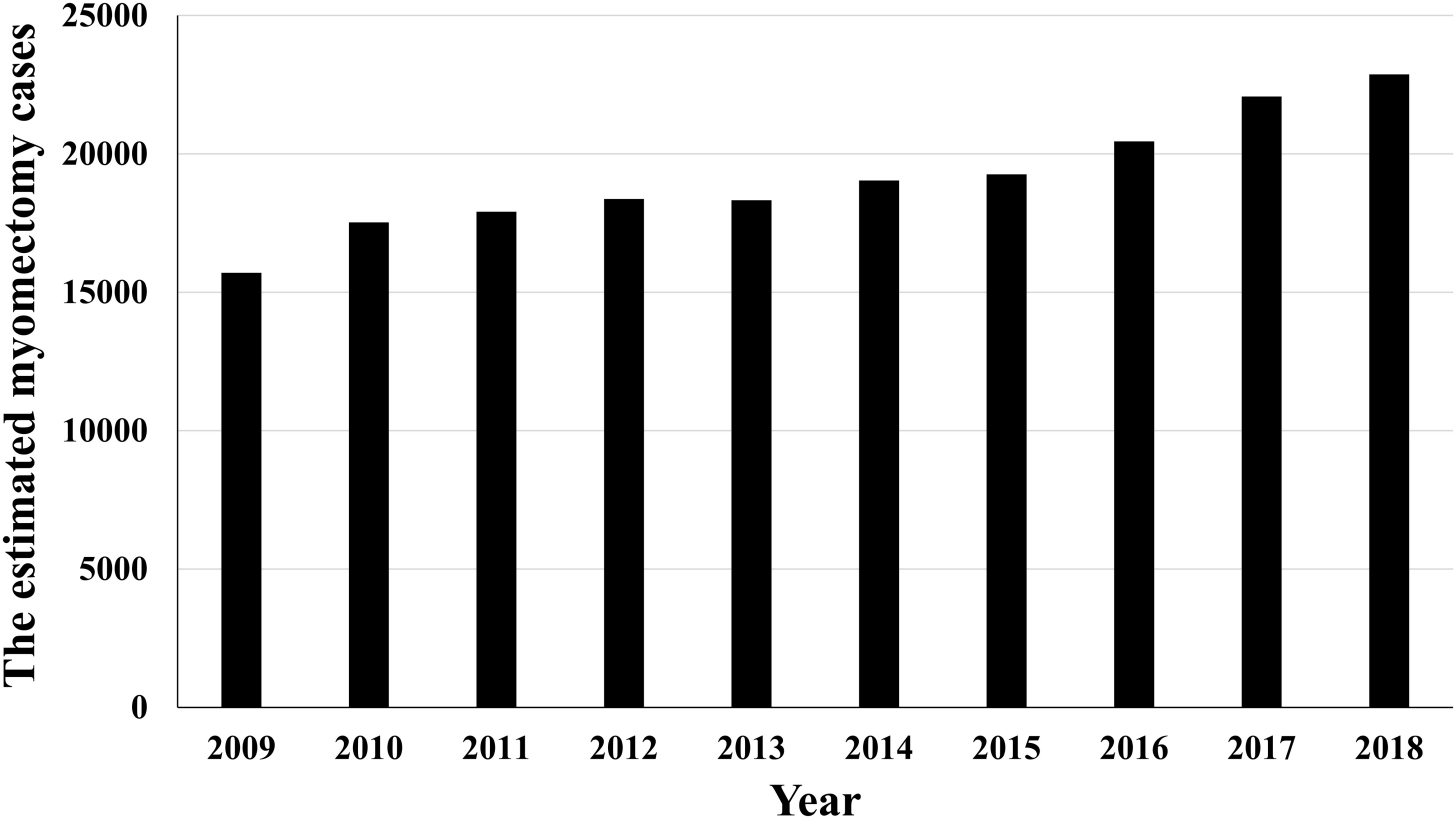
The estimated number of myomectomy cases in different years from the HIRA-NISHIRA-NIS database.

In this study, the data of 23,549 women aged 15–55 years who underwent myomectomy were extracted from a database of 3,307,214 women, recorded between 2009 and 2018 (Figure 1). The number of myomectomies performed increased annually over the years (Figure 2). The average age was 39.39 ± 0.04 years, and VTE was reported in two patients. Patient characteristics are shown in Table 2. One patient who underwent myomectomy died. However, concomitant VTE was not observed in this patient (Table 3). Among women aged 15 to 55 years, the myomectomy rate was 14.6 ± 0.1 per 10,000 patients (Table 4). The mortality rate after myomectomy was 1.3 ± 0.8 per 10,000 patients (Table 4). The incidence rates of VTE, DVT, and PE after myomectomy were 5.7 ± 1.6 per 10,000, 4.4 ± 1.4 per 10,000, and 2.5 ± 1 per 10,000, respectively (Table 4). The conversion rate to hysterectomy during myomectomy was 2.9 ± 1.1 per 10,000 patients (Table 4).

**Table 2:** Characteristics of myomectomy in HIRA-NPS 2009-2018.

	**Non-VTE**	**VTE**	***p***
	**(*N* = 23,547)**	**(*N* = 2)**	
Age	39.39 ± 0.04	42.93 ± 3.65	0.45
SES			1^a^
Mid~high SES	23,246 (98.7%)	2 (100.0%)	
Low SES	301 (1.3%)	0 (0.0%)	
Hospital grade			0.791^a^
Clinic	4,134 (17.6%)	1 (50.0%)	
Hospital	6,473 (27.5%)	0 (0.0%)	
General hospital	7,630 (32.4%)	1 (50.0%)	
Senior general hospital	5,310 (22.6%)	0 (0.0%)	
CCI			0.097^a^
0	17,857 (75.8%)	1 (50.0%)	
1	2,834 (12.0%)	0 (0.0%)	
2	2,253 (9.6%)	0 (0.0%)	
3~	603 (2.6%)	1 (50.0%)	

*CCI, charlson comorbidity index; HIRA, health insurance review and assessment service; NPS, national patient sample; SES, socioeconomic status; VTE, venous thromboembolism.*

a *Fisher's exact test*.

**Table 3:** VTE according to age per 5 years or death in myomectomy patients (HIRA-NPS 2009-2018).

	**Non-VTE**	**VTE**	***p*-value**
	**(*N* = 23,547)**	**(*N* = 2)**	
Death			1^a^
No	23,546 (100.0%)	2 (100.0%)	
Yes	1 (0.0%)	0 (0.0%)	
Age per 5 years			0.592^a^
~20	16 (0.1%)	0 (0.0%)	
~25	233 (1.0%)	0 (0.0%)	
~30	1,761 (7.5%)	0 (0.0%)	
~35	4,145 (17.6%)	0 (0.0%)	
~40	5,606 (23.8%)	1 (50.0%)	
~45	6,530 (27.7%)	0 (0.0%)	
~50	4,002 (17.0%)	1 (50.0%)	
~55	1,254 (5.3%)	0 (0.0%)	
DVT			<0.001^a^
No	23,547 (100.0%)	1 (50.0%)	
Yes	0 (0.0%)	1 (50.0%)	
PE			<0.001^a^
No	23,547 (100.0%)	1 (50.0%)	
Yes	0 (0.0%)	1 (50.0%)	

*DVT, deep vein thrombosis; HIRA, health insurance review and assessment service; NPS, national patient sample; PE, pulmonary embolism; VTE, venous thromboembolism.*

a *Fisher's exact test*.

**Table 4:** The rates or estimated cases about myomectomy in HIRA-NIS 2009-2018.

	**Rate or**	**Estimated cases**
	**incidence**	**in South Korea**
Myomectomy	14.6 ± 0.1^a^	191,514 ± 1,251
Mortality in myomectomy	1.3 ± 0.8^b^	10 ± 10
Conversion from myomectomy to hysterectomy	2.9 ± 1.1^c^	56.2 ± 21.3
VTE in myomectomy	5.7 ± 1.6^d^	17.7 ± 12.6
DVT in myomectomy	4.4 ± 1.4^d^	10 ± 10
PE in myomectomy	2.5 ± 1^d^	7.7 ± 7.7

*DVT, deep vein thrombosis; HIRA, health insurance review and assessment service; NIS, national inpatients sample; PE, pulmonary embolism; VTE, venous thromboembolism.*

*Data were expressed as mean values ± standard error.*

a *The rate of myomectomy per 10,000 women.*

b *The rate of death per 10,000 myomectomy women.*

c *The conversion rate from myomectomy to hysterectomy per 10,000 myomectomy women.*

d *The rate of disease per 10,000 myomectomy women*.

## Discussion

To our knowledge, this is the first report on the mortality rate after myomectomy from a large database of over 6 million patients. Based on data obtained from the HIRA-NIS, we found that the mortality rate after myomectomy was 0.013% among women aged 15–55 years. The mortality rate after myomectomy was considerably lower than that reported in the past. One patient who underwent myomectomy died, and this patient did not have concomitant VTE. The incidence rates of VTE, DVT, and PE after myomectomy were 0.057, 0.044, and 0.025%, respectively. The conversion rate to hysterectomy after myomectomy was 0.029%. These values were also considerably low.

Uterine leiomyomas (myomas or fibroids) are the most common type of pelvic tumors in women, with a lifetime risk of approximately 70–80% (2). There are many treatment options for leiomyoma-related symptoms, including expectant treatment, medical treatment, non-excisional procedures (magnetic resonance guided focused ultrasound, endometrial ablation, uterine artery embolization), and surgery (radiofrequency ablation, myomectomy, hysterectomy) (3). Myomectomy is performed to remove leiomyomas surgically from the uterus, keeping the uterus in place for women who wish to become pregnant or retain the uterus.

At the turn of the 20th century, compared with the mortality rate of 6–7% for abdominal hysterectomy, abdominal myomectomy was associated with a mortality rate of 40% (4–6). Hemorrhage and embolism have been the main causes of death in patients who underwent myomectomy (6). There are three possible complications associated with the operation, namely hemorrhage, postoperative morbidity, and mortality. The incidence of these complications was observed to gradually reduce with improvements in surgical techniques. Severe hemorrhage may be addressed using a number of techniques, including intraoperative blood salvage, uterine artery ligation, tourniquet application, use of vasoconstrictive agents, or conversion to hysterectomy. The mortality rate after myomectomy dropped to <1% in the 1950's (6) and was no longer higher than that after hysterectomy (6). In addition, there was no difference in complication rates between those who underwent myomectomy and hysterectomy (21).

Although myomectomy is a common operation in the field of gynecology, there are very few studies on the mortality associated with myomectomy. Therefore, we performed this study to investigate the current mortality rate of myomectomy in the era of advanced surgical skills and improved anesthesiology. In the Republic of Korea, where national health insurance is provided, most myomectomy data are acquired through the HIRA database. Therefore, we studied the mortality rate of myomectomy using the health insurance claims data. We also determined the VTE rate and conversion rate to hysterectomy. In the future, it is necessary to compare the current mortality rates of hysterectomy and myomectomy.

VTE has long been recognized as a possible complication and a leading cause of death in patients undergoing pelvic surgery (16). Pelvic operations are more prone to complications due to VTE than operations at other sites (16). PE is one of the most serious and common preventable cause of in-hospital deaths after surgery (22). Without thromboprophylaxis, the risk of DVT in patients undergoing major general or gynecologic surgery is 15–30%, and the risk of fatal PE is 0.2–0.9% (23). In a population of patients who selectively received prophylactic anticoagulants (38% of patients), the rate of VTE was 0.2% (24). Patients undergoing myomectomy (major surgery, defined as lasting for >30 min) are at a low-to-moderate risk of VTE and require appropriate thromboprophylaxis, whether mechanical or pharmacologic. In the Republic of Korea, the overall rates of postoperative VTE in major orthopedic, cancer, and benign surgeries were 1.24, 0.67, and 0.05%, respectively. Colorectal cancer surgeries (1.67%) and hip fracture (1.60%) were associated with the highest rates of VTE (25). Patients undergoing surgery for ovarian, colorectal, pancreatic, and esophageal cancers and major orthopedic surgery had a >20-fold increased risk of developing VTE than those undergoing benign surgery (25). Some authors have demonstrated that the rates of postoperative VTE in Asia, especially in the Republic of Korea, are lower than those in Caucasian populations (25). For this reason, thromboprophylaxis is not commonly offered in South Korea unless a major surgery (cancer-related surgery) is performed.

In our study, the incidence rates of VTE, DVT, and PE after myomectomy were 0.057, 0.044, and 0.025%, respectively, in the Korean population. The incidence of VTE was considerably lower than that in the general population worldwide and even in a population of patients who received prophylactic anticoagulants (VTE incidence rate: 0.2%). PE-related mortality was reported to fall from 3.3% (2001 to 2005) to 1.8% (2010 to 2013) in one study and from 17 to 10% in another study, (26, 27) with a decreasing trend each year. One of the 23,549 patients who underwent myomectomy between 2009 and 2018 died, and this patient did not have concomitant VTE. In Asian countries, such as South Korea, where VTE incidence is low, the contribution of VTE to the mortality rate associated with myomectomy is likely to be small. We believe that the considerably low mortality rates of myomectomy are due to the relatively low VTE rates in addition to improvements in surgical techniques and advanced anesthesiology methods in South Korea. However, the current study had a limitation when compared with other studies as there were no data about the body mass index and smoking status, which are often related to DVT.

Hysterectomy may be required intraoperatively to control bleeding. In our study, the conversion rate to hysterectomy during myomectomy was 0.029%. This is extremely low compared to that reported in previous studies. In cases of conversion to hysterectomy, the myomectomy code may have been omitted because there was no difference in reimbursement even if it was not entered. This could explain why the conversion rate to hysterectomy was very low in our study. Thus, future studies on the conversion rate to hysterectomy with different research methods for extracting data are required.

Myomectomy has been reported to temporarily reduce uterine volume and relieves symptoms in ~80% of the patients (28). The risk of recurrence after myomectomy is approximately 27% for single leiomyomas and >50% for multiple leiomyomas (29). If the first surgery is performed for a single leiomyoma, 11% of the women require subsequent surgery, (30) if multiple leiomyomas are removed during the initial surgery, 26% require subsequent surgery.

Our study has several limitations. First, the mortality rates in this study may be lower than the actual rate because the incidence rates in this study included only those recorded in medical institutions. Therefore, deaths outside these institutions may not have been incorporated in this study. Additionally, sudden death occurring as a side effect of myomectomy is rare. Considering the narrow land area, the density of medical institutions, relatively low medical expenses, and free emergency transportation (119) provided by the county, the ratio is likely to be very low. Second, the conversion rate to hysterectomy during myomectomy may be much lower than the actual rate because the myomectomy surgical code was not included in some cases of conversion to hysterectomy. Third, our study design did not distinguish between open and laparoscopic surgeries. However, there was only one recorded death, and the comparison of the mortality rates between laparoscopic surgery and laparotomy did not seem to be warranted. Fourth, we could not consider the risk factors, size and number of leiomyomas, surgical time etc. However, there was only one recorded death; thus, these factors were considered not significant. It is meaningful to investigate incidence rates of myomectomy-related mortality and venous thromboembolism in South Korean populations. Finally, the cause of death was unknown but was not related to cancer or trauma due to an accident.

In conclusion, we found that the mortality rate after myomectomy in South Korea is considerably lower than that reported in previous studies, especially at the turn of the 20^th^ century. The myomectomy-related DVT rate is also lower than that reported in previous studies, especially in studies conducted in the Caucasian population. We have established that the current mortality rate after myomectomy has markedly reduced and that the incidence of DVT is low in this Asian population.

## Data Availability Statement

The datasets presented in this study can be found in online repositories. The names of the repository/repositories and accession number(s) can be found at: https://figshare.com/articles/dataset/Myomectomy_mortality/14157521.

## Ethics Statement

This retrospective chart review study involving human participants was conducted in accordance with the Ethical Standards of the Institutional and National Research Committee and with the 1964 Helsinki Declaration and its later amendments or comparable ethical standards. The Institutional Review Board (IRB) of Sanggye Paik Hospital of Inje University granted an official waiver of ethical approval and informed consent (IRB approval number: SGPAIK 2020-07-006).

## Author Contributions

J-SY and MK: project development, data collection, statistical analysis, and manuscript writing. Both authors contributed to the article and approved the submitted version.

## Funding

This work was supported by the Inje University research grant (20150896).

## Conflict of Interest

The authors declare that the research was conducted in the absence of any commercial or financial relationships that could be construed as a potential conflict of interest.

## Publisher's Note

All claims expressed in this article are solely those of the authors and do not necessarily represent those of their affiliated organizations, or those of the publisher, the editors and the reviewers. Any product that may be evaluated in this article, or claim that may be made by its manufacturer, is not guaranteed or endorsed by the publisher.
